# Characterization of Immunogenicity Associated with the Biocompatibility of Type I Collagen from Tilapia Fish Skin

**DOI:** 10.3390/polym14112300

**Published:** 2022-06-06

**Authors:** Jingyi Zhang, Jeevithan Elango, Shujun Wang, Chunyu Hou, Meng Miao, Jia Li, Lixin Na, Wenhui Wu

**Affiliations:** 1 College of Public Health, Shanghai University of Medicine & Health Sciences, Shanghai 201318, China; zhangjyj@163.com; 2 Department of Marine Bio-Pharmacology, College of Food Science and Technology, Shanghai Ocean University, Shanghai 201306, China or jelango@ucam.edu (J.E.); ytxiaoyu@163.com (C.H.); 3 Department of Biomaterials Engineering, Faculty of Health Sciences, UCAM-Universidad Católica San Antonio de Murcia, 30107 Murcia, Spain; 4 Co-Innovation Center of Jiangsu Marine Bio-Industry Technology, Jiangsu Ocean University, Lianyungang 222005, China; shujunwang86@163.com; 5 College of Medical Technology, Shanghai University of Medicine & Health Sciences, Shanghai 201318, China; diguoazu@163.com (M.M.); violet_629@163.com (J.L.)

**Keywords:** type I collagen, immunogenicity, biocompatibility

## Abstract

Collagen from fish has been proven to have a low antigenicity that has no difference in the genetic codes compared with mammalian-based collagen. This study was designed to investigate the impact of tilapia skin collagen on immunogenicity and biocompatibility in vivo and in vitro. The structural characteristics of both acid-soluble and pepsin-soluble collagen (ASC and PSC), determined using SDS-PAGE and atomic force microscopy imaging experiments, revealed that the collagen had the basic characteristics of type I collagen (COL-I). The in vitro biocompatibility of the collagens showed good cell proliferation against human foreskin fibroblast (HFF-1) cells. PSC and ASC were considered to be almost non-hemolytic biomaterials with favorable blood compatibility in hemolysis tests. The in vivo antigenicity of the collagen in an ICR mouse model evoked an acceptable specific inflammatory response compared to bovine collagen. The implant’s position had developed a complete granulation tissue and the sponge disappeared after 8 weeks. The level of cytokines produced by the COL-I immune response was much lower than bovine collagen, which indicated the appropriate implantable property and biodegradability of the collagens. In conclusion, the tilapia COL-I has a lower immunogenicity with better compatibility than bovine COL-I and is a potential alternative to conventional mammalian collagens in biomedical uses.

## 1. Introduction

Among natural biomaterials, collagen has become the preferred one in tissue engineering applications [1]. It is considered a proper candidate for tissue-engineered scaffolds due to its low antigenicity and excellent biocompatibility [2,3,4,5]. Collagen is a ubiquitous natural material in the human body, and it is responsible for maintaining the structural integrity of many organisms. Type I collagen (COL-I) is the most abundant extracellular matrix protein in mammals. It acts as the mechanical structural support for bone, skin, tendons, ligaments, and blood vessels, and plays an important role in extracellular cues for regulating physiological processes including cell adhesion, proliferation, and differentiation. Collagen has been used in numerous applications: in drug delivery, soft tissue augmentation, and suturing, and as a skin substitute and tissue engineering substrate [6,7]. COL-I has been extensively used as a biomaterial for the development of tissue engineering constructs and wound dressing systems due to its low antigenic and high direct cell adhesion properties [8,9,10].

However, collagen is present not only in mammals, but also throughout the entire animal kingdom, including birds and fishes. The main sources of COL-I are still limited to bovine, porcine, and chicken [11]. With the outbreak of zoonotic infectious diseases, such as bovine spongiform encephalopathy (BSE), the use of mammalian-derived COL-I for scientific research or food supplements has become questionable [12]. Another issue that confines the use of mammalian collagen is an allergic feature, as evidenced by a previous report [13]. Furthermore, in countries having religious restrictions, the application of certain mammalian-animal-isolated products is strictly prohibited. Marine organisms could be a valuable collagen source that is widely available, with no risks of disease transmission. Within marine resources, collagen is commonly isolated from fish skins [14,15]. Fish skin can be obtained from food industries, supporting the availability of fish collagen in abundance. Each year, thousands of tons of ocean fish are destined for human consumption, generating a considerable number of byproducts that are usually discarded as commercial waste. Tilapias are now the most important farmed fish globally, with a total production of 5.6 million tons in 2018 [16]. It is possible to obtain abundant COL-I extract from tilapia skin.

In general, the macromolecular features of a protein are more likely to encourage an immune response than shared features with the host species. Thus, the application of collagen from aquatic animals needs to be realized on the immunological response, biocompatibility, and other safety concerns such as antigenicity and immunogenicity. Indeed, even though many collagen-based products have proved highly effective in clinical use [17,18], in some cases for several decades, recurrent questions continue to be asked regarding the immunological safety of collagen products. Thus, before it is confirmed to be safe to use as a possible biomaterial, the COL-I from tilapia skin should be validated by certain immunological data in tissue engineering applications. In these clinical studies [19,20,21], both type I collagens sensitization and antibody responses have been reported that have largely reflected the early animal data on the immunogenicity of tilapia collagens.

In our previous study, we evaluated the structural characteristics and toxicity of collagen for biomedical applications [22]. The microstructure of the collagen was analyzed using circular dichroism, Fourier transform infrared, and scanning electron microscopy. The toxicity response was tested with an acute systemic toxicity assessment. The results indicated that tilapia skin COL-I had no toxicity responsivity and had a potential value for biomedical applications. Tilapia skin could be qualified as a good source of collagen due to the abundant protein content in the raw material and its easy extraction. The current study aims to identify further biocompatibility issues including response evaluation by antigenicity and subcutaneous implantation in vivo. Cytotoxicity and hemolysis tests were also performed to check the cytocompatibility and blood compatibility with collagens.

## 2. Materials and Methods

### 2.1. Extraction of Type I Collagen from the Skin of Tilapia

The skins were instantly denuded, washed with distilled water, and cut into small pieces (2 × 8 mm), then frozen at −20 °C, within a week before collagen extraction. The tilapia skin was homogenized using a tissue homogenizer with a phosphate buffer (pH 6.5). All procedures were carried out at 4 °C. The homogenate was treated with double distilled water at the ratio of 1:6 (*w*/*v*) for 24 h to remove water-soluble substances. Furthermore, the skin was decalcified with 0.5 M ethylenediamine tetraacetic acid at the ratio of 1:10 (*w*/*v*) for 48 h.

Acid-soluble collagen was isolated from the pretreated skin according to the methods of Zhang et al., Matmaroh et al., and Jeevithan et al. [22,23,24,25] with suitable modification. The pretreated tilapia skin was soaked in 0.5 M acetic acid (1:6, *w*/*v*) for 4 days with continuous shaking and the extract was centrifuged at 10,000 rpm for 30 min at 4 °C. Acid-soluble collagen was extracted twice with 0.5 M acetic acid for 3 days and the resultant precipitates were harvested through centrifugation. The supernatant was collected and salted out by adding 2 M NaCl. The precipitates were re-dissolved in a minimum volume of 0.5 M acetic acid and dialyzed against distilled water for 2 days until a neutral pH was obtained. The dialyzed sample was lyophilized and referred to as acid-soluble collagen (ASC). Pepsin-soluble type I collagen was isolated from the tilapia skin according to the method of Chen et al. [26].

### 2.2. SDS-Polyacrylamide Gel Electrophoresis and Atomic Force Microscopy Imaging

Collagen purity was examined using SDS-polyacrylamide gel electrophoresis (SDS-PAGE). SDS-PAGE was performed using a discontinuous Tris-HCl/glycine buffer system, 7.5% separating gel, and 5% stacking gel. After electrophoresis, the gels were visualized with Coomassie Brilliant Blue R-250, and the decolorization of the gels was performed with a methanol-distilled water-acetic acid solution [27,28].

In order to investigate well-characterized collagen, atomic force microscopy (AFM) was used in our study. AFM images of the collagen films were obtained in the air using a microscope. PSC and ASC were prepared with a thickness of 10 μm. The samples were mounted on AFM metal discs, while locator grids were used to map the surface so that the same area of collagen could be selected exactly. The topographic AFM images are presented on a color scale that represents the Z height. Observations from each sample were taken from several locations, but only the most representative are illustrated. The surface images used scan widths of 1 μm × 1 μm.

### 2.3. Cytotoxicity Analysis

HFF-1 cells were offered by Shanghai Zhong Qiao Xin Zhou Biotechnology Co., Ltd. Adherent cells that grew out from the skin were harvested through trypsin treatment and used as skin fibroblasts. Cells were maintained in Dulbecco’s modified Eagle’s medium (DMEM) with 15% fetal bovine serum (FBS) at 37 °C in a CO_2_ incubator (Shanghai Hengyue Medical Instruments Co., Ltd., Shanghai, China).

The toxicity of the collagen from tilapia skin can be initially assessed using the standard MTT (3-(4,5-Dimethylthiazol-2-yl)-2,5-diphenyltetrazoliumbromide) method. The OD value can reflect the survival rate of the cells. Standard bovine collagen (T-I), pepsin solubilized (PSC), and acid solubilized collagens (ASC) were coated in microtiter plates (96-well, Costar) overnight at 4 °C with different concentrations (1, 10, 50, and 100 μg/mL). The control consisted of uncoated wells. Cells were then added at 5 × 10^4^ cells/mL and incubated at 37 °C for 2 days. The medium was removed from the wells in 96-well plates by gentle sucking. MTT solution (0.5 mg/mL in PBS) was added to each well and incubated for 4 h. After that, the MTT solution was removed from the well and 100 μL/well DMSO was added to solubilize the formazan crystal. Detection and quantification of the cytotoxicity were performed using a multi-well plate reader at 570 nm.

### 2.4. Hemolysis Test

Hemolysis testing of prepared hydrogels was performed as reported before according to ISO 10993-4 (15) [29]. A total of 1.5 mL of blood from the ear vein of the New Zealand rabbit was collected in the triangle bottle of glass beads for the removal of fibrin by slight stirring. The blood was poured into a centrifugal 10 mL saline tube and centrifuged at 2500 rpm/min for 5 min. The supernatant was discarded and repeated three times until a transparent supernatant was obtained following the steps above. Normal saline water (0.9 *w*/*v* NaCl) was added to obtain red cells made of 2% cell suspension solution.

The samples were cut into a size of 0.5 cm × 1 cm with a thickness of 0.5 mm, and 20 mL of normal saline water (0.9 *w*/*v* NaCl) was added to each sample and incubated at 37 °C for 72 h. The positive control and the negative control consisted of a 2% cell suspension solution in distilled water and a 2% cell suspension solution in normal saline water (0.9 *w*/*v* NaCl), respectively. All the samples and controls were placed at a constant temperature of 37 °C in an incubator for 3 h.

The supernatant was drawn using a UV-visible spectrophotometer at the wavelength of 545 nm to measure the absorbance value of the tube, and the hemolysis rate was calculated. The samples were considered toxic if they had a greater than 5% hemolytic percentage. The percentage hemolysis was calculated using the following relationship:

Percentage hemolysis (%) = (OD of experimental sample − OD of negative control sample)/(OD of positive control sample − OD of negative control sample) × 100.

### 2.5. Immunological Analysis

All the animal study protocols and procedures were approved by the Institutional Ethics Committee of Shanghai Ocean University OF Institute (Ethical Approval No. SHOU–DW–2022–053).

The animals were maintained in an air-conditioned animal house under controlled environmental conditions of humidity (50–70%), temperature maintained at 25 ± 2 °C, and 12 h: 12 h light/dark cycle. The animals had access to standard feed and water ad libitum. All laboratory animals were housed in polypropylene cages with a wire mesh top and a hygienic bed of husk.

ICR female mice were divided into 8 groups with different treatments. The collagen treatment groups were injected with an intraperitoneal injection on days 1, 7, and 14 with a 0.1 mL collagen solution (PSC and ASC) at different concentrations (1, 10, and 100 μg/mL). The control group was injected with an intraperitoneal injection on days 1, 7, and 14 with 0.1 mL 0.1 M acetic acid. The positive control group was injected with an intraperitoneal injection of bovine tendon collagen (T-I) (100 μg/mL) on days 1, 7, and 14. All the injections were repeated at intervals of 7 days. Then, blood tests were taken 7 days after each immunization 3 times. The immunogenicity (short-term) of collagen was evaluated in a mice model in terms of collagen total antibody content (Mouse COL-I Ab) and IgG, IgA, and IgM, respectively.

### 2.6. Subcutaneous Implantation

Six male rabbits (8–9 weeks) received implants of tilapia collagen sponge (PSC and ASC) and bovine tendon collagen sponge. Bovine tendon collagen sponge was obtained from Beijing Yierkang Bioengineering Company Limited as the control group. Anesthesia was induced with an intravenous injection of Nembutal (30 mg/kg body weight) via the ear vein. A total of 2 cm on either side of the spine of the rabbit’s skin was closely shaved and swabbed with 75 % alcohol. Six small transverse incisions were made in the skin on both sides of the equidistance from the rabbit spinal, an extending pocket was dissected bluntly beneath the panniculus carnosus, and a square of collagen sponge (1 cm × 1 cm) was inserted into the pocket and the incision closed by surgical suture. Anti-infection was conducted by injecting ceftriaxone sodium 2 times a day, and lasts for 3 days after implantation.

Two rabbits (one for PSC and T-I implantation, another for ASC and T-I implantation) were killed each 1, 4, and 8 weeks after implantation. The dorsal skin with the attached sponges and the muscles beneath was excised. Tissues were fixed with a 10% formaldehyde solution, and then the fixed tissue was embedded in paraffin sectioned at 3 μm and stained with hematoxylin and eosin following standard techniques [30]. Sections were observed for two surrounding inflammatory reactions of the test and the formation of the lumen of the fibers. For analysis with enzyme-linked immunosorbent assays (ELISA), tissue near the implant position was obtained from each experimental animal and each tissue fluid in the distilled water (*w*/*v* = 1:3) at 4 °C until use.

### 2.7. Statistical Analysis

The complete experiment was performed in duplicate with three samples of the same condition analyzed for each assay (*n* = 3 for all data). Statistical analysis was carried out using an unpaired Student’s *t*-test. A value of *p* < 0.05 was considered to be statistically significant.

## 3. Results

### 3.1. SDS-Polyacrylamide Gel Electrophoresis and Atomic Force Microscopy

**Figure 1:**
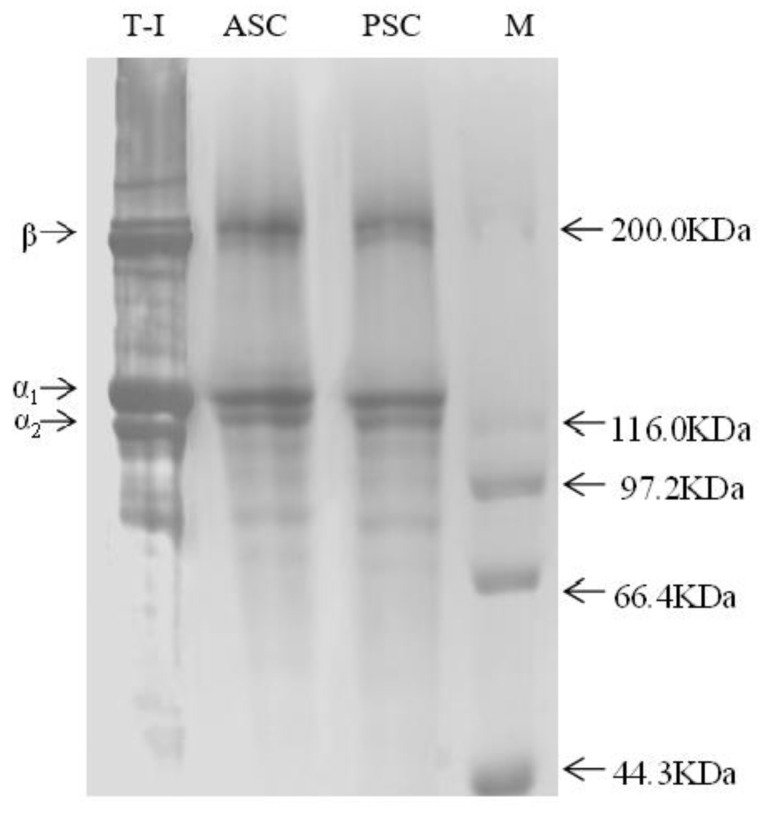
Protein pattern of collagens from SDS-PAGE.

The SDS-PAGE results (Figure 1) showed that ASC and PSC had similar trends in protein band patterns. Moreover, the results were similar to those of bovine collagen (T-I), which accorded with the characteristic composition of type I collagen. A similar purity of the collagen extracts was shown by electrophoresis, confirming the reliability of the extraction process.

**Figure 2:**
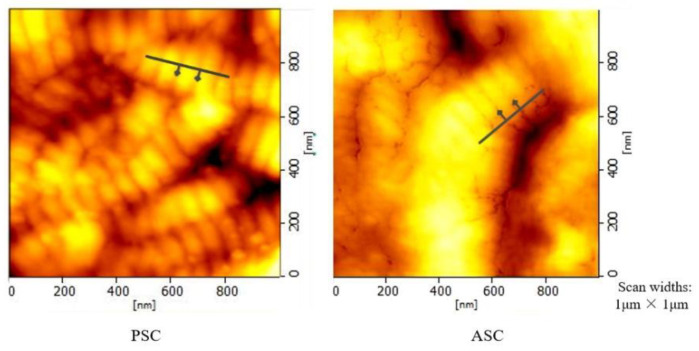
AFM-Microstructural image of tilapia collagen.

The characterization of collagen was performed using atomic force microscopy (AFM) imaging [31,32]. This feature can be used for measuring the characteristic D-band periodicity of collagen fiber. An area of a collagen thin film (where the D-band of several collagen fibrils was observed) was selected for monitoring D-band periodicity alterations. As Figure 2 demonstrates, the AFM image of a collagen fibril with a D-band and the height profile showed a periodicity of 78.27 nm (PSC) and 67.17 nm (ASC). Interestingly, tilapia collagen retains the fiber arrangement of intact collagen, which forms fiber bundles [33]. The results showed that the collagen from tilapia skin obtained through enzymatic hydrolysis and acid hydrolysis extraction had a relatively complete collagen fiber structure.

### 3.2. Cytotoxicity Analysis

**Figure 3:**
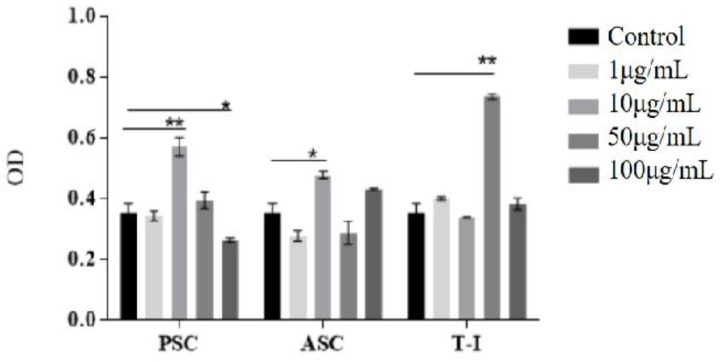
Viable cells OD at 570 nm of different concentrations of collagens (control: without collagen; PSC: tilapia PSC; ASC: tilapia ASC; T-I: bovine collagen). All values are mean + SD (*n* = 3). Different superscripts in the same column indicate significant differences compared with the control group (* *p* < 0.05; ** *p* < 0.01).

Cytotoxicity was detected through an MTT assay to inspect the viability of the HFF-1 grown with PSC and ASC. The OD value of MTT was positively correlated with cell survival. HFF-1 fibroblasts proliferated continually with respect to the culture time for all samples. As depicted in Figure 3, the number of viable cells was significantly higher in some collagen-coated wells than in the control (uncoated) well. It can be seen that the cell viability of the test sample (1 μg/mL) was comparable to that of the control group, except for ASC. After that, it was increased with a 10 μg/mL collagen sample PSC and ASC. PSC and ASC have positive effect on cell viability in four cases of collagen concentration, i.e., 10 μg/mL of PSC (0.57) 50 μg/mL of PSC (0.39), 10 μg/mL of ASC (0.47) and 100 μg/mL of ASC (0.43). The viability of T-I and control were similar (0.33–0.38). T-I had a beneficial effect on the cells at 50 μg/mL collagen concentration (0.74). These data demonstrate a significant difference in the percentage viability of cells grown with collagen groups compared with the control group.

These results indicate that none of the COL-I extracted from tilapia skin in the study was harmful to the proliferation capacity, and the viability of HFF-1 was more suitable for fibroblast adhesion and growth at a suitable concentration.

### 3.3. Hemolysis Test

In vitro blood compatibility was evaluated through the hemolytic percentage of the synthesized samples. Table 1 shows the percentage hemolysis of all the samples. The positive control had noticeable hemolysis, while the PSC and ASC groups were non-hemolytic. The saline group was similar to the collagen group, for which there was no obvious hemolysis. The hemolysis safety standards calculation result of the hemolysis rate by collagen absorbance values in the PSC, ASC, and bovine tendon collagen were 1.29%, 0.99%, and 2.73%, respectively. All the samples were non-hemolytic because they are within the allowed range (i.e., <5%).

### 3.4. Immunological Analysis

The levels of pro-inflammatory cytokine secretions and antibody secretions were measured here to determine whether tilapia collagen induces any inflammatory response comparable to that induced by bovine collagen.

**Figure 4:**
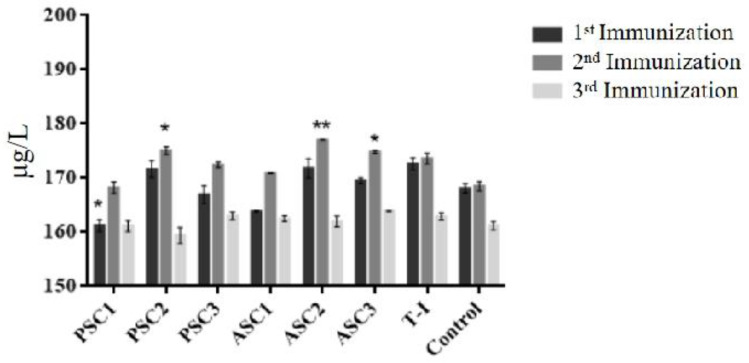
The mass concentration of tilapia collagen antibodies in mice. All values are mean + SD (*n* = 3). Different superscripts in the same column indicate significant differences compared with the control group (* *p* < 0.05; ** *p* < 0.01).

All the mice produced antibodies in the antigenicity test against tilapia collagen as antigen. As shown in Figure 4, for the first and second immunizations, the antibody concentration of ASC2 (10 μg/mL) produced the highest immunoreaction and was followed by the group of PSC2 (10 μg/mL), with the lowest immune response being PSC1 (1 μg/mL). During the third immunization, ASC3 (100 μg/mL) became the highest response group, similar to PSC3, followed by ASC1 and PSC1 (1μg/mL), and the lowest immune response was PSC2 (10 μg/mL). There was no significant difference in the control and T-I groups in the production of collagen antibodies between the first and second immunization. When compared with the control during the first immunization, the difference was not significant except for the processing of PSC1 (1 μg/mL) (*p* < 0.05). PSC2 (*p* < 0.05), ASC2 (*p* < 0.01), and ASC3 (*p* < 0.05) had a significant difference with the control group in the second immunization. There was no significant difference between each group after the third immunization.

The test sera were comparable with those of the acetic acid control group and the T-I control groups. According to the cylindrical diagram, the total antibody response of 1 μg/mL PSC after the first immunizations with sera collection over 7 days was minimal according to ELISA. Moreover, the antibody response of the ASC group at 10 μg/mL was increased compared with that of all the other groups. After 21 days, the difference was not significant, which may have been due to the immune adaptability of mice to collagen after three immunizations. After the third immunization, the antibody content in all injection groups decreased to the lowest level and remained at 159.39 μg/L–163.92 μg/L.

In this experiment, three easy-to-detect immunoglobulin indexes were tested in order to observe the antigenicity. Even after three immunizations (21 days), the total Ig response was lower according to ELISA (Table 2). The 10 μg/mL tilapia collagen group had a higher ELISA absorbance compared with the 100 μg/mL and 1 μg/mL tilapia collagen groups. In comparison, the levels of ASC2 from IgG were higher than those of ASC1 and ASC3 in the treatment group. The levels of IgG, IgA, and IgM in the groups of PSC1, PSC3, ASC1 and ASC3 were lower than those in the control group. The mice treated with T-I were highly responsive to tilapia collagen when the concentration was 100 μg/mL and 1 μg/mL, but the response was similar between 10μg/mL collagen and T-I. The groups of 100 μg/mL and the groups of 1 μg/mL have no significant difference. The standard deviation (SD) value analysis shows that the distinction absorbance of each mouse between the average and SD values was negligible. The ability of the protein content to influence the production of immunoglobulin over a continuous period requires further study for a comprehensive understanding.

### 3.5. Subcutaneous Implantation

#### 3.5.1. Macroscopic Observation

**Figure 5:**
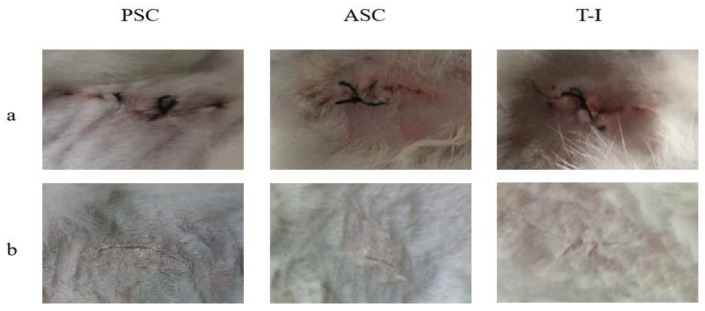
The surface of New Zealand rabbit skin observed (**a**) immediately after the implantation, and (**b**) after 8 weeks.

The surgical operations were successful, and no death or infection occurred after the surgery. As shown in Figure 5, all New Zealand rabbit back surgery incisions healed well with no complications. In the surrounding tissues, there was no bleeding, swelling, or abnormal secretions. Rabbit activity decreased slightly after implantation, including declines in spirit and appetite, but recovery occurred the next day. During the 8-week implantation schedule, the rabbits showed no signs of general or local health problems.

In the first week after implantation, the implant was bonded completely with the cells. In the position of the implant, no bleeding or swelling were observed. As shown in Figure 5, the implants become gradually smaller in a time-dependent manner. After 8 weeks, the position of the implant developed a complete granulation tissue and the sponge disappeared completely. The in vivo test employed here was effective for evaluating the inflammatory response of the implant itself because of minimal surgical damage around the implanted site.

#### 3.5.2. Histological Assessment

**Figure 6:**
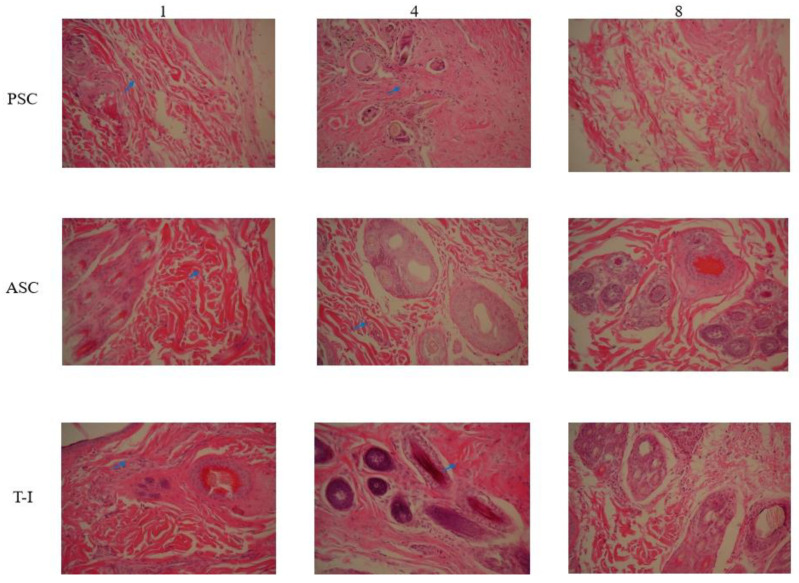
HE staining slice of New Zealand rabbit skin at 1st-, 4th-, and 8th-week cross-section 200 X.

A histological analysis of the implantation experiment was performed using HE staining. From the histological evaluation (Figure 6), the collagens were completely degraded over 8 weeks without any foreign body reaction, which indicates acceptable biocompatibility and biodegradability. The degradation performed well, making it suitable for tissue engineering. The histopathological evaluation of the T-I group at 1 week and 4 weeks showed hemorrhages and inflammatory cell infiltrations (blue arrow). The tissue sections of the PSC and ASC groups showed a significant reduction in inflammatory cells in comparison to the T-I group at 8 weeks. Rejuvenation of skin appendages was evident at 8 weeks in all groups. The inflammatory reaction was significantly lower in the animals of all groups at 8 weeks in comparison to the groups at 1 week and 4 weeks. It seems that PSC and ASC showed better results when compared to the T-I group. All the treatments with collagen regimens significantly enhanced the fibrocytes (as the mature mesenchymal cells). Overall, the healing condition of the group treated with tilapia collagen was more similar to that of natural skin, and it had the best cosmetic appearance, with a normal thickness of the epidermal layer and rejuvenation of the hair follicles and skin appendages.

#### 3.5.3. Biochemical Parameters

**Figure 7:**
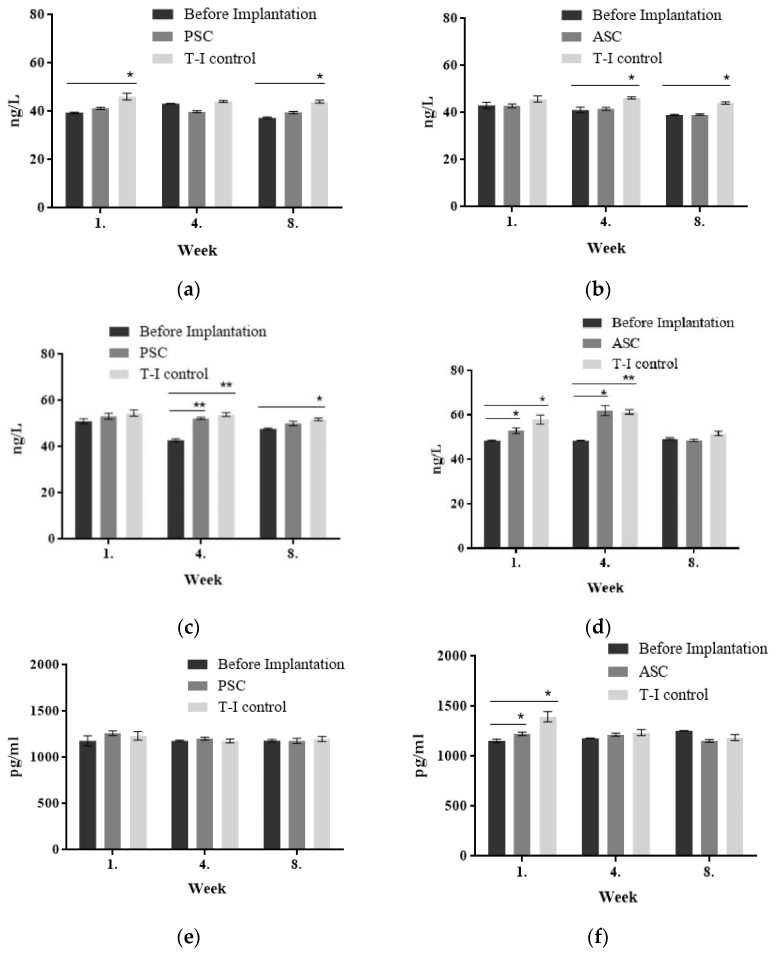
The levels of IL-4, IL-6, TGF-β, and TNF-α in the implantation experiment. (**a**) IL-4 content of PSC implantation; (**b**) IL-4 content of ASC implantation; (**c**) IL-6 content of PSC implantation; (**d**) IL-6 content of ASC implantation; (**e**) TGF-β content of PSC implantation; (**f**) TGF-β content of ASC implantation). All values are mean + SD (*n* = 3). Different superscripts in the same column indicate significant differences compared with the control group (* *p* < 0.05; ** *p* < 0.01).

The biocompatibility of a subcutaneously implanted tilapia collagen sponge was studied in rabbits by analyzing the level of antibodies using ELISA methods. After the rabbits were implanted with tilapia collagen and bovine collagen, the levels of IL-4, IL-6, and TGF-β in the T-I control group were increased due to the inflammatory reaction (Figure 7). There was no significant change (*p* > 0.05) in the level of IL–4 between PSC (39.61–41.28 ng/L) and ASC (39.19–42.94 ng/L) in the implanted animals. On the other hand, these levels were significantly lower than in the T-I control group (43.64–46.14 ng/L). The level of IL-6 was lower than in the T-I control group in the same period from the first to the eighth week, and both two groups displayed a decreasing trend as time progressed. In the PSC-implanted group, the content of TGF-β was 1263.33 pg/mL in the first week, which was a little higher than that in the T-I control group, which was 1234.16 pg/mL. Then, it gradually declined to 1180.00 pg/mL during the eighth week. The level of TGF-β in the PSC- and ASC-implanted group maintained the same downward trend after the implantation. The levels of these three pro-inflammatory factors decreased gradually over time in New Zealand rabbits.

After subcutaneous implantation, the tilapia collagen could stimulate and activate immune cells such as lymphocytes and mononuclear macrophages in vivo in rabbits, making it an immune regulator of inflammatory cytokines upon secretion of IL-4, IL-6, and TGF-β. In this sense, tilapia collagen produced a certain immune response after implantation into New Zealand rabbits. The changes in these data resemble those found in the results from the previous antigenicity experiment. When collagen was used in the organism as an exogenous substance, the inflammatory reaction in animals would maintain for several weeks, and then keep stable until the collagen was completely absorbed. The results indicated that tilapia collagen could produce an immune response after being implanted in the rabbit. Three kinds of pro-inflammatory factors declined gradually over time (Figure 7). The levels of IL-4, IL-6, and TGF-β produced by the tilapia collagen immune response were much lower than those for bovine tendon collagen during the experiment period.

## 4. Discussion

The results of the SDS-polyacrylamide gel electrophoresis and atomic force microscopy show that the collagen from tilapia skin had a relatively complete collagen fiber structure. Collagen with the characterization of such models offers the advantage of providing a physiologically relevant environment for culturing cells.

When compared to the control group in the cytotoxicity analysis, the percentage of HFF-1 cell proliferation was increased with a 10 μg/mL collagen concentration. The results show that the total cell viability of fibroblasts treated with both fish and mammalian collagen scaffolds was rapidly increased compared to control cell lines (without any treatment) in a suitable concentration, and a pronounced effect was achieved with ASC and PSC. Therefore, the porous biomaterial can promote fibroblast proliferation [34].

It was easier for fibroblasts to adhere to and become uniformly distributed on the PSC and ASC, which could facilitate the interaction between cells and collagens [35]. Therefore, in vitro tests are used for studying tissue cell toxicology, and a complete understanding of in vivo cell responses is required for successful implantation in tissue engineering.

Compared to the collagen hemolysis study in mice [36], the hemolysis rate of PSC and ASC in our study was much lower than 2%. Moreover, in a study of collagen hydrogel [37], the collagen product had a hemolysis index of 1.19 %. The contact of biological materials with blood can influence homeostasis locality. Then, it can induce a series of adverse biological reactions, such as hemolysis, coagulation, and inflammation. Therefore, the excellent properties of collagen provide a basic guarantee of good blood compatibility, with a significantly reduced adverse impact on patients. The rate of hemolysis of PSC and ASC did not cause a hemolytic reaction, which proved the good blood compatibility. A favorable blood compatibility was the primary condition for biomedical materials in contact with blood. The collagen sponges were within acceptable limits. Collagen extracted from marine by-products was a perfect matrix applied in biomedical aspects.

It was believed that a lower production of the inflammatory factor by biomaterials could be a desirable factor for suitable usage in biomedical applications. When compared to bovine collagen, the percentage of inflammatory contents was acceptable with the addition of ASC and PSC, which showed the better biocompatibility properties of those implantation materials.

Host reactions following biomaterial implantation include injury, blood–material interactions, acute or chronic inflammation, and granulation tissue development. Depending on the extent of an injury, the acute inflammatory response to biomaterials at the implant site usually occurred quickly, within a few days. Then, chronic inflammation developed at the implant site characterized by the presence of mononuclear cells (i.e., monocytes and lymphocytes) [38]. The presence of chronic inflammatory responses for a period of longer than 3 weeks usually indicates an infection. After the chronic inflammation stage, new healthy tissue could be identified in the granulation tissue characterized by the presence of macrophages, fibroblast infiltration, and neovascularization. The immunogenic behavior (short-term immunity) of collagen was evaluated by measuring immunoglobulins, IgG, IgA, and IgM, and the results proved that there was not much difference in the level of immunoglobulins between collagen and the control group.

Bassetto et al. [39] found in a histological study that collagen-treated wounds developed healthy granulation tissue, with increased blood vessels, and underwent progression with a high proliferation index eventually towards stable tissue. A few studies have been carried out on the immunological fate of implanted collagen sponges such as bovine collagen. In these investigations, the collagen implants were tolerated and were progressively degraded and replaced by granulation tissue, which was similar to the tilapia collagen. Even though all the sponges induced a transient inflammatory response before their degradation, the intensity of immunization was acceptable.

As reported by Hiroaki [40], pellet implantation tests into the paravertebral muscle of rabbits demonstrated that tilapia collagen caused rare inflammatory responses during 1- and 4-week implantations similar to those of porcine collagen and a high-density polyethylene as a negative control. In this study, the levels of IL-4, IL-6, and TGF-β of the collagen-treated groups were lower than those of the T-I control group, suggesting that tilapia collagen has a lower subcutaneously implanted immunogenicity than commercial (mammalian) collagen products. In this way, we recognized that tilapia collagen may serve as a kind of raw material that can exist for a long time in an organism without producing a severe immune response. Chuanglong et al. [41] used an indirect ELISA method to detect rabbit serum antibody levels after the implantation of raw material for bone glue, and found a low immune response. This also supports the present findings.

## 5. Conclusions

In summary, our findings indicate the potential of tilapia collagen as an alternative to mammalian collagen in biomedical uses. The tilapia skin collagen sponge produced with vacuum freeze-drying technology has more effective biological properties owing to its better absorption of tissue exudates, its promotion of fibroblast and epithelial cell growth, and its generation of granulation tissue [42,43,44]. In this study, all collagen sponges possessed good blood compatibility, which could be applied in biomedical material fields. Cytotoxicity indicated that tilapia collagen exhibited higher cell viability than naturally derived biomaterials (T-I). For the whole experiment, tilapia collagen exhibited a comparable effect to bovine collagen. The content of inflammatory factors produced by the immunological reaction of tilapia collagen was significantly lower than that of bovine tendon collagen, which indicated that the tilapia collagen had a higher latent capacity than the common bovine tendon protein as better implants. The implantation test showed that ASC and PSC had better biocompatibility properties compared to the control group. The in vivo test employed here was effective for evaluating the inflammatory response of the implant itself because of minimal surgical damage around the implanted site. As the use of kinds of mammalian collagen may be restricted in the future due to BSE, foot, and mouth disease, the current work suggests that the COL-I derived from tilapia, usually an underutilized material, offers promise as an alternative to mammalian collagen and may be useful for fibrocyte regeneration. Our further study will explore the potential of tilapia collagen in orthopedic surgeries, where collagen is widely used as a filming material for promoting skin regeneration and as a scaffolding material for skin and bone tissue engineering.

## Figures and Tables

**Table 1:** Hemolysis testing results.

Serial No	Sample ID	OD at 545 (nm)	% Hemolysis
1	PSC	0.041 ± 0.00047 *	1.29 ± 0.047 *
2	ASC	0.038 ± 0.0014 *	0.99 ± 0.13 *
3	TI	0.056 ± 0.0023	2.73 ± 0.23

* All values are mean + SD (*n* = 3). Different superscripts in the same column indicate significant differences compared with the control group (T-I) (*p* < 0.05).

**Table 2:** The Ig concentration of mice treated with tilapia skin collagen.

Samples	IgG (ng/mL)	IgA (μg/mL)	IgM (ng/mL)
PSC1	429.01 ± 3.91	47.94 ± 0.43	1.82 ± 0.01 *
PSC2	437.59 ± 3.39	49.53 ± 0.80	1.89 ± 0.04
PSC3	424.81 ± 3.02	46.86 ± 0.34 *	1.81 ± 0.01 *
ASC1	429.50 ± 3.05	47.99 ± 0.44	1.82 ± 0.01 *
ASC2	440.03 ± 2.14 *	49.90 ± 0.34	1.89 ± 0.02
ASC3	425.06 ± 3.09	47.02 ± 0.54	1.81 ± 0.01 *
T-I	435.59 ± 2.81	48.83 ± 0.35	1.87 ± 0.01
Control	429.53 ± 3.04	48.58 ± 0.33	1.87 ± 0.02

* All values are mean + SD (*n* = 3). Different superscripts in the same column indicate significant differences compared with the control group (*p* < 0.05).

## Data Availability

All data included in this study are available upon request by contacting the corresponding author.
